# Influence of *Pediococcus pentosaceus* Starter Cultures on Biogenic Amine Content and Antioxidant Activity in African Sourdough Flatbread Fermentation

**DOI:** 10.3390/antiox13101204

**Published:** 2024-10-07

**Authors:** Alaa Ahmed Alsiddig Hassan, Young Hun Jin, Jae-Hyung Mah

**Affiliations:** Department of Food and Biotechnology, Korea University, Sejong 30019, Republic of Korea; alaa16@korea.ac.kr (A.A.A.H.); younghoonjin3090@korea.ac.kr (Y.H.J.)

**Keywords:** fermented sorghum, starter culture, lactic acid bacteria, BA production, BA degradation, DPPH scavenging activity, total phenolic content

## Abstract

This study investigated the impact of *Pediococcus pentosaceus* strains not only on biogenic amine (BA) content, but also on antioxidant indices, including 2,2-diphenyl-1-picrylhydrazyl (DPPH) scavenging activity and total phenolic content, in kisra, an African sourdough flatbread. Among forty-six lactic acid bacteria (LAB) strains isolated from naturally fermented kisra sourdough, two strains (K-B21, K-B01) identified as *P. pentosaceus,* were selected due to their low BA-producing and high BA-degrading ability for kisra fermentation. Inoculation with *P. pentosaceus* K-B21 or *P. pentosaceus* K-B01 completely prevented the formation of tyramine and cadaverine during kisra fermentation. The levels of putrescine, histamine, spermine, and spermidine in kisra were reduced by about 90%, >31%, 55–61%, and 9–25%, respectively, by the two strains, compared to the control (natural fermentation). Additionally, DPPH scavenging activity was 83–84% in the control and inoculated groups of kisra. The total phenolic content was 1977.60 μg/g in the control and insignificantly lower in the inoculated groups (1850–1880 μg/g) than the control. These results suggest that *P. pentosaceus* K-B21 and K-B01 are promising candidates for use as sourdough starter cultures to produce kisra bread of higher quality, including both its safety and health functionality.

## 1. Introduction

Sourdough is traditionally used to improve flavor, nutritional value, texture, and shelf-life in bakeries. Sourdough is an acidic or sharp-tasting fermented mixture of flour and water for making bread from cereals flour [1]. Due to its microbial life, sourdough is metabolically active [2] and used as a starter culture to inoculate newly prepared dough [3]. Sourdough has been successfully used in the production of highly nutritional, value-added bread with a good sensory profile [4]. The unique organoleptic quality and characteristic of sourdough bread arises from the indigenous microflora, which is primarily represented by distinctive lactic acid bacteria (LAB) [5]. Sourdough fermentation can occur in both firm dough or liquid suspension [2], and the acidity of the dough depends on its physical properties [6]. The liquid suspension type of sourdough is widely practiced in northern African countries. It has been used to produce a form of flat bread that is traditionally made and consumed on a daily basis. In Sudan, sourdough bread known as kisra is made of water and sorghum (*Sorghum bicolor*) or millet (*Pennisetum typhodium*) flour mixture inoculated by previously fermented dough.

Traditional production of kisra relies on spontaneous fermentation by LAB. Although both LAB and yeasts have been isolated from spontaneously fermented kisra [7,8], LAB are dominant microorganisms in the food [7,9]. When kisra undergoes spontaneous fermentation, the process is difficult to control due to the diverse microbial community, which may lead to the production of biogenic amines (BAs) by undesired microbes. BAs are low-molecular-weight nitrogen compounds that occur naturally [10] in various foods and are involved in various biological activities in most living organisms. Despite their bioactive roles, some BAs in food are toxic and mainly produced by microbial decarboxylation of amino acids [11]. Several LAB species demonstrate decarboxylase activity, which leads to the formation of BAs in lactic fermented foods [12]. The occurrence of such BAs was also found in kisra [13].

Lactic fermentation has been recognized as a traditional processing method for enhancing the nutritional values of foods and preserving various phytochemicals that contribute to the antioxidant properties of the final products [14]. Likewise, kisra, a lactic-fermented sorghum food [7], is considered a valuable source of dietary antioxidants [15]. This is probably due to both the fermentation process and the use of sorghum flour rich in polyphenols [16]. According to a previous study [17], combining fermentation with baking can enhance the antioxidant indices in kisra, including antioxidant activity and the content of flavonoid, phenolic, and tannin. However, to date, no research has investigated the impact of utilizing starter cultures in kisra fermentation on the antioxidant indices of kisra.

Due to the very complex microbial composition of the naturally fermented kisra, it is necessary to carefully select strains to be used as starter cultures, capable of improving food safety, health functionality, and microbial performance. The objective of this study was to inhibit the formation of BAs during kisra fermentation by employing autochthonous LAB with low BA-producing and high BA-degrading abilities as starter cultures. Given the growing consumer interest in fermented food products with antioxidant potential, this study also assessed the effect of the non-BA-producing LAB on the antioxidant indices of kisra when used as starter culture.

## 2. Materials and Methods

### 2.1. Analysis of Bacterial Production Capability of BAs by LAB Strains

A total of forty-six presumably LAB strains previously isolated from kisra sourdough [13] were assessed for their BA-producing ability. The ability of the strains to produce BAs was evaluated according to the previous studies with modifications [18,19]. The strains were twice activated into de Man, Rogosa, and Sharpe (MRS, Laboratories Conda Co., Madrid, Spain) broth and incubated at 37 °C for 48 h. Each strain was then inoculated at 0.1% (*v*/*v*) into MRS broth supplemented with 0.005% (*w*/*v*) pyridoxal-5-phosphate and each of the following precursor amino acids at 0.5% (*w*/*v*): L-ornithine hydrochloride, L-tyrosine, L-histidine hydrochloride monohydrate, and L-lysine monohydrochloride (all from Sigma-Aldrich Chemical Co., St. Louis, MO, USA). The final pH of the broths was adjusted to 5.80 using 2 M hydrochloric acid. After incubation at 37 °C for 48 h, the concentration of BAs in the MRS broth were measured, as described in Section 2.8.

### 2.2. Identification of Non-BA Producers

Based on the BA production ability of each LAB strain, the two strains K-B21 and K-B01 with weak BA-producing ability were selected for kisra fermentation (Table 1). Identification of the selected two strains at the species level was performed through 16S RNA sequencing analysis. The 16S rRNA was obtained through PCR amplification using the following universal bacterial primers pair: 518F (5′-CCAGCAGCCGCGGTAATACG-3′) and 805R (5′-CCCCCAGCCTAGCTTAGTTT-3′) (SolGent, Daejeon, Republic of Korea). Identification of bacterial strains was conducted through BLAST searching.

### 2.3. Analysis of Toxic BA Degradation Ability of the Selected P. pentosaceus Strains

Degradation of toxic BAs, histamine and tyramine, by the selected *P. pentosaceus* strains was determined following the procedures described by Lee et al. [20]. Briefly, 10 µL glycerol stock of each selected strain was inoculated in 5 mL MRS broth. After incubation at 37 °C for 48 h, a loopful of the culture was streaked on MRS agar and incubated under the same conditions. A single colony was transferred into 5 mL MRS broth. After incubation, 200 µL of the culture was inoculated in 10 mL MRS broth to obtain a sufficient number of bacterial cells and then incubated under the same conditions along with shaking at 200 rpm. Then, the culture was centrifuged at 9000× *g* for 10 min at 4 °C. After discarding supernatant, the bacterial cells were washed twice with 0.05 M sodium phosphate buffer (pH 7.00; 4.0962 g Na_2_HPO_4_ and 2.5370 g NaH_2_PO_4_ dissolved in 1L distilled water; all from Sigma). The bacterial cells were resuspended in 10 mL of the same buffer but containing 2 toxic BAs, 0.5 mM histamine and 0.5 mM tyramine (*w*/*v*; all from Sigma). The bacterial cell suspension was incubated with shaking at 200 rpm for 24 h at 30 °C. Then, the buffer was filtered through a 0.2 µm membrane filter (Millipore Co., Bedford, MA, USA) and was subjected to BA analysis (see Section 2.8; hereafter, the filtrate is referred to as “sample buffer”) The same buffer without bacterial cells served as blank, called “blank buffer”. The degradation rate of toxic BAs was calculated as follows: degradation rate (%) = [1 – (C_sample_ / C_blank_) × 100], where C_blank_ is the concentration of each toxic BA in the blank buffer, and C_sample_ is that in the sample buffer.

### 2.4. Cell Culture and Microbial Inoculation

Cell cultures were prepared following the procedure described by Sinnelä et al. [21], with minor modifications, including MRS broth and incubation condition (37 °C, 48 h) for the enrichment of the selected *P. pentosaceus* strains. Each bacterial inoculum was then subsequently added to the dough (prepared in Section 2.5.1) at approximately 1%, with the final concentration adjusted to 10^7^ CFU/g. Kisra fermentation was categorized into four groups: the K-B21 group, inoculated with *P. pentosaceus* K-B21; the K-B01 group, inoculated with *P. pentosaceus* K-B01; the blank group, sorghum flour sterilized and non-fermented without an inoculum; and the control group, which was naturally fermented and served as the control.

### 2.5. Kisra Preparation

#### 2.5.1. First Fermentation (Sourdough—Ajin)

**Figure 1:**
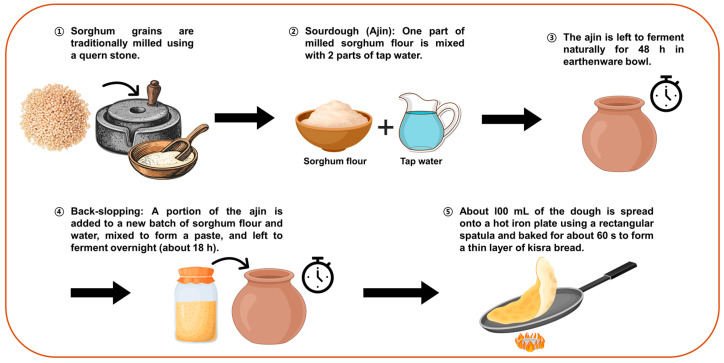
Schematic illustration of traditional kisra processing.

Figure 1 shows a schematic illustration of traditional kisra processing. In this study, kisra preparation followed the method described by Mohammed et al. [7]. Briefly, a starter sourdough (ajin) was prepared under sterile conditions by mixing 100 g of sorghum flour (Bob’s Red Mill, Milwaukee, OR, USA) with 200 mL of sterile distilled water. For the K-B21 and K-B01 groups, each bacterial suspension prepared above was inoculated. For the blank and control groups, the same amount of sterile distilled water instead of the bacterial suspension was added. Unsterilized sorghum flour was used for the control group; whereas, sorghum flour sterilized at 100 °C for 45 min was used for the blank and inoculated groups. The sourdough was fermented at 37 °C for 48 h and sampled aseptically at 0, 24, and 48 h.

#### 2.5.2. Second Fermentation (Back-Slopping)

For the second fermentation, about 30 g of the sourdough starter was back-slopped into a fresh mixture of 100 g sorghum flour (as in the previous section) and 200 mL sterile distilled water. The mixture was fermented at 37 °C for 12 h and sampled aseptically at 0, 6, and 12 h. At the end of fermentation, a small amount of the fermented dough was spread over a hot plate (150–160 °C), forming a very thin flake within 60 s.

### 2.6. Physicochemical Properties

The physicochemical properties were determined as follows: The pH was measured with a pH meter (Thermo Scientific, Waltham, MA. USA), and total titratable acidity (TTA) was determined by the method of AOAC [22] and expressed as a percentage of lactic acid (each 1 mL of 1 N NaOH is equivalent to 90.08 mg of lactic acid). Water activity (a_w_) was determined using a water activity meter (AquaLab Pre; Meter Group, Inc., Pullman, WA, USA).

### 2.7. Bacterial Counts

Decimal dilutions were prepared by homogenizing 5 g of sample with 45 mL of 0.1% sterile peptone water using a vortex (Vortex-Genie, Scientific Industries, Bohemia, NY, USA). The LAB counts were enumerated on MRS agar at 37 °C for 48 h. Counts were expressed as the logarithm of colony-forming units per milligram (log CFU/g).

### 2.8. Analysis of BAs in Kisra Samples and Bacterial Cultures

The preparation of sample extracts and standard solutions, chromatographic separation, and derivatization of BAs were performed according to the method described by Ben-Gigirey et al. [18,19]. Standard solution (tryptamine, β-phenylethylamine hydrochloride, putrescine dihydrochloride, cadaverine dihydrochloride, histamine dihydrochloride, tyramine hydrochloride, spermidine trihydrochloride, and spermine tetrahydrochloride all from Sigma) spiked with 1,7-diaminoheptane (1 mg/mL; Sigma) was as internal standard.

### 2.9. Antioxidant Indices Assays

#### 2.9.1. DPPH Free Radical Scavenging Activity

The 2,2-diphenyl-1-picrylhydrazyl (DPPH, Alfa Aesar, Haverhill, MA, USA) radical scavenging assay was performed according to the procedure previously described by Braca et al. [23]. Briefly, 1 mL of the sample methanolic extract (1:10, *w*/*v* dilution) was mixed with 1 mL of freshly prepared methanolic DPPH solution (0.004%) and, then, incubated in the dark for 30 min. The absorbance was read at 517 nm using a spectrophotometer (Lambda 35; PerkinElmer Ltd., Waltham, MA, USA). The antioxidant activity was expressed as a percentage of inhibition.

#### 2.9.2. Total Phenolic Content (TPC)

Total phenolic content was determined by the Folin–Ciocalteu modified method [24]. In summary, 150 µL of the sample methanolic extract (1:10, *w*/*v* dilution) or gallic acid dilutions (Samchun Pure Chemical Co., Ltd., Pyeongtaek, Republic of Korea) was added to 750 µL of Folin–Ciocalteu reagent (50% [*v*/*v*] dilution with deionized water, Sigma). After standing at room temperature for 3 min, 600 µL of 2% Na_2_CO_3_ was added to the mixture and kept in the dark for 30 min. The standard curve was plotted based on gallic acid concentration (R^2^ = 0.996). Total phenolic content was expressed as gallic acid equivalent per g of wet samples (GAE/g).

### 2.10. Statistical Analysis

The results were analyzed using one-way analysis of variance (ANOVA) using the Minitab statistical software, version 17 (Minitab Inc., State College, PA, USA). The compatibility of variable distribution was verified with the Fisher LSD pairwise comparison test at 5% probability (*p*), and *p* < 0.05 was considered statistically significant. Each measurement was taken three times, and the fermentation experiments were carried out in two separate trials.

## 3. Results and Discussion

### 3.1. Selection and Identification of LAB Strains as Starter Candidates Isolated from Kisra Sourdough

In a previous study, a total of forty-six LAB strains were isolated from naturally fermented kisra sourdough [13]. Among them, two strains were selected based on low BA production ability. Through further in vitro BA degradation tests for the two strains, they had a high degradation ability of histamine and tyramine, the most toxic BAs among the eight BAs commonly found in fermented kisra (Table 1). Specifically, the K-B21 strain displayed no histamine production and the second-lowest tyramine production (0.69 ± 0.02 µg/mL), and the K-B01 strain exhibited the lowest tyramine production (0.63 ± 0.01 µg/mL) and the second-lowest histamine production (0.19 ± 0.01 µg/mL). Also, the K-B21 strain had degradation rates of 8.06 ± 5.86% and 8.38 ± 9.01% for histamine and tyramine, respectively, the K-B01 had low degradation rates of 3.28 ± 4.64% and 6.20 ± 6.33% for histamine and tyramine, respectively. Both LAB strains were identified as *P. pentosaceus* by 16S rRNA gene sequencing and applied for kisra fermentation to inhibit BA accumulation in the food.

During kisra fermentation, the microbial population mainly consisted of *Lactobacillus*, *Lactococcus*, *Pediococcus*, and *Enterococcus* [8,25,26]. Mohammed et al. [7] reported *P. pentosaceus* dominated until the end of first fermentation and during the consecutive secondary fermentation. Furthermore, *P. pentosaceus* has been reported to effectively inhibit the formation of BAs during cucumber fermentation [27] and wine making [28]. Considering these previous reports, the use of the strains selected in this study may, therefore, ensure the microbiological stability of sourdough and mitigate the BA-associated risks of sourdough bread, including kisra.

### 3.2. Effect of P. pentosaceus K-B21 and P. pentosaceus K-B01 on Physiochemical and Microbial Properties during Kisra Fermentation

**Figure 2:**
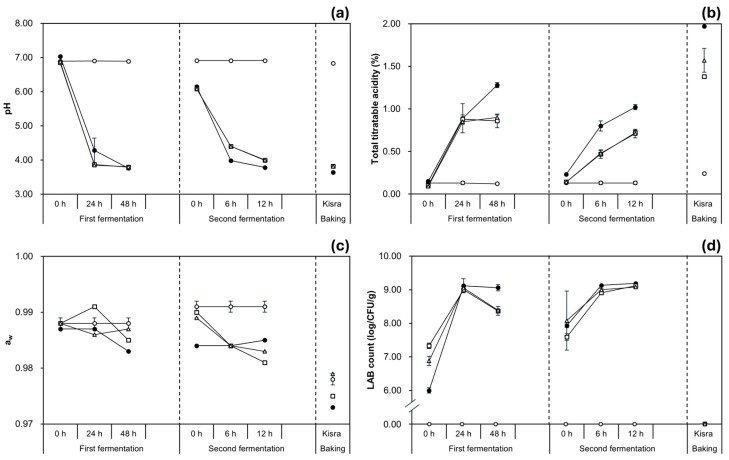
Changes in the (**a**) pH, (**b**) total titratable acidity, (**c**) water activity, (**d**) LAB count during kisra fermentation with the selected *P. pentosaceus* strains. ○: blank group (kisra non-fermented), ●: control group (kisra naturally fermented without an inoculum), □: K-B21 group (kisra inoculated with *P. pentosaceus* K-B21), ⧍: K-B01 group (kisra inoculated with *P. pentosaceus* K-B01). Error bars indicate standard deviations calculated from triplicate experiments.

To unveil the fermentation dynamics of kisra inoculated with the selected *Pediococcus* strains, the changes in physiochemical (pH, TTA, and a_w_) and microbial (LAB count) properties during kisra fermentation were analyzed, as shown in Figure 2 and Table S1. In the first fermentation, the initial pH was similar across all groups (blank, control, and two inoculated groups), ranging from 6.85 to 7.03. While the pH value of the blank group stayed constant during fermentation, those of the other groups decreased to the range of 3.76 to 3.80, when measured at 48 h. The initial TTA values ranged from 0.09% to 0.15% in all the groups. At 48 h, the control group marked the highest TTA value of 1.28 ± 0.03%, while the TTA values of both the K-B21 and K-B01 groups remained constant at 0.90 ± 0.08% and 0.86 ± 0.03%, respectively. The TTA value of the blank group remained constant during fermentation. The initial LAB count was about 6.0 log CFU/g in the control group, while those in the inoculated groups were about 7.0 log CFU/g, respectively. The LAB counts then increased to ≥9.0 log CFU/g at 24 h in all three groups. Thereafter, the count of the control group remained statistically constant until 48 h. However, the counts of the K-B21 and K-B01 groups decreased slightly but significantly (*p* < 0.05) to about 8.4 log CFU/g, respectively. In the blank group, LAB were not detected throughout the fermentation. The a_w_ values during the first fermentation ranged from 0.983 to 0.991 in all the groups.

The second fermentation showed similar initial pH values across all groups fermented by LAB (control, K-B21, and K-B01 groups), ranging from 6.08 to 6.15. At 12 h, the pH values of the K-B21 and K-B01 groups were about ≤4.0, while the control group had a significantly lower pH of 3.78 ± 0.00. The initial pH value of the blank group was 6.91 ± 0.01, and the value remained constant during fermentation. The initial TTA values of all groups ranged from 0.13% to 0.23%. Subsequently, at 6 h, the TTA value significantly increased to 0.80 ± 0.06% in the control group, while both the K-B21 and K-B01 groups experienced a modest increase to around 0.47%. Similar trends were observed at 12 h, with the TTA value reaching 1.02 ± 0.03% in the control group, which is significantly (*p* < 0.05) higher than approximately 0.72% in both the K-B21 and K-B01 groups. The TTA value of the blank group stayed constant during fermentation. The initial LAB counts ranged from 7.59 to 8.08 log CFU/g across all fermentation groups. At 12 h, the LAB counts increased in all the fermentation groups reaching 9.00–9.20 log CFU/g, with no significant differences (*p* > 0.05). In the blank group, LAB were not detected throughout the fermentation. The a_w_ ranged from 0.991 to 0.981 in all groups, during the second fermentation.

After baking, the pH decreased sharply to 6.83 ± 0.01 in the blank group, 3.64 ± 0.00 in the control group, 3.82 ± 0.01 in the K-B21 group, and 3.81 ± 0.01 in the K-B01 group. Meanwhile, the TTA values increased significantly to 0.24 ± 0.01% in the blank group, 1.97 ± 0.00% in the control group, 1.38 ± 0.01% in the K-B21 group, and 1.57 ± 0.14% in the K-B01 group. Baking also resulted in a complete elimination of LAB count in all the groups. Additionally, the a_w_ values decreased significantly after baking, ranging from 0.973 to 0.979 in all the groups. In addition, the use of *P. pentosaceus* K-B21 and K-B01 strains as starter cultures effectively led to roughly similar physicochemical and microbial changes to the natural fermentation, contributing to successful kisra bread production.

Despite the similarity of the properties between all the groups, there were some differences between the control and inoculated groups in the detailed changes. During each of the first and second fermentations, the LAB counts of the inoculated groups were lower than the control, which can be explained by previous reports of Jideani et al. [29] and Luti et al. [30]. Jideani et al. [29] suggested that a decrease in LAB count may occur as fermentation progresses because the LAB reach the stationary phase. In contrast, the presence of indigenous microbes such as yeasts, different from LAB, breaks down the starch into nutrients that LAB can easily metabolize, which maintains the steady exponential phase of the LAB population, as reported by Luti et al. [30]. Meanwhile, although there was no significant difference in pH between the control and inoculated groups, the TTA was much higher in the control than in the inoculated groups. This difference may be because the indigenous LAB present in the control group produce more lactic acid than the selected LAB strains, and the further acidification by them may increase their BA production as a homeostatic response to neutralize the low environmental pH [12,31,32]. Therefore, the reports explain the changes not only in microbial properties but also in physicochemical properties in the fermentation experiments of this study (i.e., lower LAB count, higher pH, and lower TTA in the inoculated groups than in the control).

### 3.3. Effect of P. pentosaceus K-B21 and P. pentosaceus K-B01 on BA Content during Kisra Fermentation

**Figure 3:**
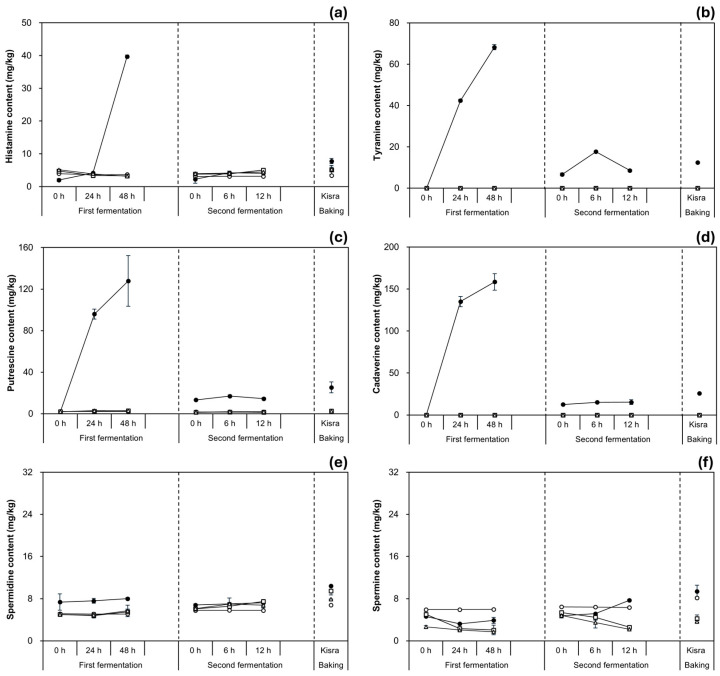
Inhibitory effects of *P. pentosaceus* K-B21 and *P. pentosaceus* K-B01 strains on BA content during kisra fermentation. (**a**) Histamine, (**b**) tyramine, (**c**) putrescine, (**d**) cadaverine, (**e**) spermidine, and (**f**) spermine. ○: blank group (kisra non-fermented), ●: control group (kisra naturally fermented without an inoculum), □: K-B21 group (kisra inoculated with *P. pentosaceus* K-B21), ⧍: K-B01 group (kisra inoculated with *P. pentosaceus* K-B01). Error bars indicate standard deviations calculated from triplicate experiments.

To evaluate the inhibitory effect of *P. pentosaceus* K-B21 and K-B01 on BA accumulation during kisra fermentation, changes in BA content were observed throughout the processing stages, as shown in Figure 3 and Table S2. The application of the two strains completely inhibited the formation of tyramine and cadaverine at all the stages. Additionally, the utilization of the strains effectively reduced the content of putrescine, histamine, spermine, and spermidine at the stages, excluding 12 h of the second fermentation, when the histamine and spermidine levels were similar to the control. Tryptamine and β-phenylethylamine were not detected in any of the blank, control, and two inoculated groups. Hereafter, BA content in the blank group was not described for the following reasons: (i) the levels of all BAs remained constant throughout the first and second fermentation; (ii) although the BA content slightly increased after baking, the levels were lower than 10 mg/kg.

Meanwhile, the changes in BA content in the inoculated groups were distinctively different from those in the control group. In the first fermentation, the control group had a substantial (*p* < 0.05) increase in histamine, tyramine, putrescine, and cadaverine content from the initial values of approximately ≤2.0 mg/kg of each BA to 39.68 ± 0.47, 68.21 ± 1.28, 127.81 ± 24.38, and 158.45 ± 9.73 mg/kg, respectively, at 48 h. In contrast, the content of these BAs in the inoculated groups showed little change, which demonstrated the inhibitory effects of the strains. Specifically, the histamine content in both the K-B21 and K-B01 groups decreased from an initial content of approximately 5.0 mg/kg to around 3.0 mg/kg, at 48 h. The putrescine content in the K-B21 and K-B01 groups remained significantly lower (<3.0 mg/kg) than the control at 48 h. Additionally, no tyramine and cadaverine were detected in all the inoculated groups.

In the second fermentation, the control group exhibited a somewhat moderate increase in histamine and cadaverine content. Histamine content increased from an initial 2.24 ± 1.21 to 4.08 ± 0.10 mg/kg, at 48 h, while cadaverine increased from an initial 12.58 ± 0.61 to 15.31 ± 2.91 mg/kg, at 48 h. Though tyramine and putrescine content were relatively stable throughout the second fermentation compared to the first fermentation, their content significantly peaked at 6 h, reaching approximately 17.0 mg/kg for both BAs. Interestingly, in the K-B21 and K-B01 groups, the changes of histamine and putrescine content followed a similar pattern to those in the control group. In both the K-B21 and K-B01 groups histamine content increased slightly from an initial about 3.0 mg/kg to roughly 5.0 mg/kg at 12 h, displaying no difference from the values in the control. Although putrescine content in the K-B21 and K-B01 groups fluctuated slightly, the content (<2.0 mg/kg of both BAs, at 12 h) was significantly lower than the control group. Tyramine and cadaverine were not detectable in the inoculated groups.

After baking, the histamine, tyramine, putrescine, and cadaverine content in the control group significantly increased to 7.78 ± 0.81, 12.39 ± 0.03, 25.40 ± 5.18, and 25.68 ± 0.78 mg/kg, respectively. The histamine and putrescine content in the inoculated groups also slightly increased after baking; however, despite the similar patterns observed during the second fermentation, the content of these two BAs remained at significantly lower levels (about 5.0 mg/kg of histamine and <3.0 mg/kg of putrescine) than in the control group. After baking, no tyramine and cadaverine were detected. Inoculated groups produced final kisra bread with significantly lower BA content, resulting in a substantial reduction in histamine (34.30% in K-B21 group, 31.60% in K-B01 group) and putrescine (89.50% in K-B21 group, 90.00% in K-B01 group) and a complete inhibition of tyramine and cadaverine (100% in both K-B21 and K-B01 groups).

In addition, unlike the dynamic change profiles of other BAs in the control group, spermidine and spermine displayed subtle variations; however, the content remained at relatively stable levels below 8.0 mg/kg in both the first and second fermentation stages. The content of spermidine and spermine in the K-B21 and K-B01 groups were <7.0 mg/kg and <3.0 mg/kg, respectively, throughout fermentation, which was slightly lower than those in the control but not significantly different. After baking, both spermine and spermidine levels increased significantly in all three groups, with the highest levels observed in the control group. The final kisra bread of K-B21 and K-B01 groups contained significantly lower spermidine (reduction of 9.17% and 26.77%, respectively) and spermine (reduction of 56.30% and 61.04%, respectively) compared to the control.

Taking the above results together, during all the processing stages, the highest amounts of BAs appeared in the control, suggesting that the presence of BA-producing LAB may promote BA formation [13,33]. In contrast, utilizing *P. pentosaceus* K-B21 and K-B01 as starter cultures in kisra production completely prevented the formation of tyramine and cadaverine (100%) at each processing stage and significantly reduced histamine, putrescine, spermidine, and spermine content. Such results are aligned with a study by Świder et al. [34] who reported the adequate elimination of tyramine and putrescine during cucumber fermentation using *P. pentosaceus* KKP 3273 isolated from tomato. Špička et al. [35] also reported a significant decrease in cadaverine content in sauerkraut inoculated with *Lactiplantibacillus plantarum* CCM 3769 compared to spontaneous fermentation. Similarly, Lee et al. [10] observed a significant histamine reduction content in miso fermented with *L. plantarum* D-103 compared to the uninoculated control. Meanwhile, in the current study, *P. pentosaceus* K-B21 showed a relatively higher histamine reduction than *P. pentosaceus* K-B01, which was likely due to its higher histamine degradation ability, as mentioned earlier (see Section 3.1). This assumption is supported by a previous report by Lee et al. [20], which showed that histamine- and tyramine-degrading LAB not only successfully reduces tyramine formation, but also significantly reduces the formation of other BAs in kimchi fermentation.

### 3.4. Effect of P. pentosaceus K-B21 and P. pentosaceus K-B01 on Antioxidant Indices during Kisra Fermentation

**Figure 4:**
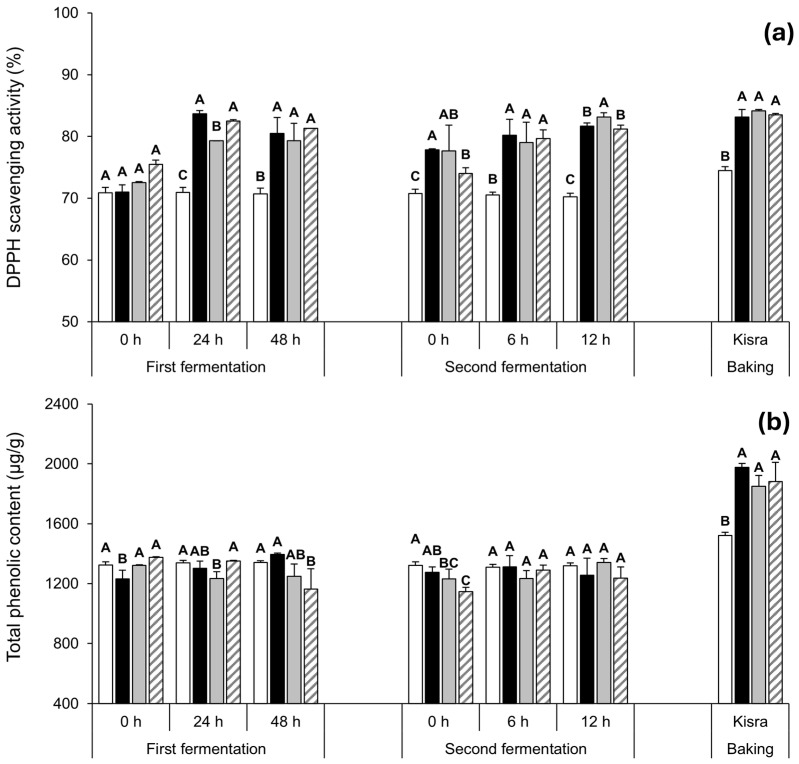
Changes in the (**a**) DPPH radical scavenging activity and (**b**) total phenolic content during kisra fermentation with the selected *P. pentosaceus* strains. □: blank group (kisra non-fermented), ■: control group (kisra naturally fermented without an inoculum), 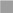: K-B21 group (kisra inoculated with *P. pentosaceus* K-B21), ▨: K-B01 group (kisra inoculated with *P. pentosaceus* K-B01). Values of bars in the same period of kisra processing with different letters (A–C) are significantly different (*p* < 0.05). Error bars indicate standard deviations calculated from triplicate experiments.

To evaluate the health functionality of *P. pentosaceus* K-B21 and K-B01, the antioxidant indices including DPPH radical scavenging activity and total phenolic content were analyzed during kisra processing (Figure 4 and Table S3). Overall, the antioxidant indices (especially DPPH scavenging activity) of kisra bread were improved through fermentation with *P. pentosaceus* K-B21 or K-B01 strains, which was similar to the control processed based on natural fermentation. This is consistent with a previous report [13]. In contrast, those of the blank group stayed constant throughout the fermentation, indicating that microbial activity is necessary for increasing the antioxidant indices.

Meanwhile, the changes in the antioxidant indices in the inoculated groups at each stage of the kisra manufacturing process were similar to those in the control group, with no significant difference. In the first fermentation, the DPPH scavenging activity in the control group increased from an initial value of 71.00 ± 7.10 to 80.50 ± 2.60% at 48 h, and the total phenolic content increased from an initial value of 1230.60 ± 58.20 to 1395.30 ± 8.30 μg/g at 48 h. Accordingly, the DPPH scavenging activity in the inoculated groups increased from approximately 75.0% of each group to 79.33 ± 2.80% of the K-B21 group and 81.33 ± 0.00% of the K-B01 group at 48 h, with no marked difference from the control. The total phenolic content in the K-B21 and K-B01 groups slightly decreased from the initial values of 1321.80 ± 4.02 and 1374.70 ± 4.20 μg/g to 1248.20 ± 83.20 and 1162.90 ± 137.30 μg/g, respectively, at 48 h, with no notable differences from the control. Initial DPPH radical scavenging activity and total phenolic content in the blank group were 70.88 ± 0.87% and 1325.10 ± 19.72 µg/g, respectively, and remained constant during the first fermentation.

In the second fermentation, the DPPH scavenging activity in the control group further increased to 81.67 ± 0.50% at 12 h; whereas, the total phenolic content slightly decreased to 1257.10 ± 112.30 μg/g at the same fermentation period. The DPPH scavenging activity in the K-B21 and K-B01 groups also further increased to 83.17 ± 0.70% and 81.17 ± 0.70% at 12 h, with no significant difference from the control. However, the total phenolic content in the K-B21 and K-B01 groups increased to 1342.40 ± 25.00 and 1236.50 ± 74.90 μg/g, respectively, at 12 h, which was opposite to the first fermentation. As for the blank group, DPPH radical scavenging activity and total phenolic content remained constant without increasing even during the second fermentation.

After baking, the DPPH scavenging activity and the total phenolic content in the control group significantly increased to 83.17 ± 1.20% and 1977.60 ± 25.00 µg/g, respectively. Similarly, both the DPPH scavenging activity (84.17 ± 0.20% in the K-B21 group and 83.50 ± 0.20% in the K-B01 group) and total phenolic content (1851.20 ± 25.00 µg/g in the K-B21 group and 1880.60 ± 128.90 µg/g in the K-B01 group) also increased in the inoculated groups after baking, with no significant difference from the control. Without exception, such antioxidant indices in the blank group increased to 74.47 ± 0.65% and 1522.24 ± 19.81 µg/g, respectively, but were significantly lower than those in all groups fermented by LAB (*p* < 0.05).

Sorghum flour has been reported to have various antioxidants and polyphenols [36,37]. In this study, at 0 h of the first fermentation, all fermentation groups (control, K-B21, and K-B01 groups) had initial DPPH radical scavenging activity and total phenolic content, ranging from 71.00–75.50% and 1230.60–1374.70 µg/g, respectively. Even such indices were also observed in the blank group (70.88% and 1325.10 µg/g, respectively), in which sorghum flour was sterilized but not fermented. It is noteworthy that the blank, K-B21, and K-B01 groups using sterilized sorghum flour had a relatively higher total phenolic content (1321.80–1374.70 µg/g) than the control group (1230.60 µg/g). Such observation may be due to the sterilization process of sorghum flour. Ofosu et al. [38] suggested that wet cooking may increase the accessibility of polyphenol compounds in sorghum.

Meanwhile, the DPPH radical scavenging activity in all groups fermented by LAB increased during the first and second fermentation; whereas, in the blank group, it remained constant during the same period. After baking, the activity increased in all the groups, but the increase was greater in the fermentation groups. This indicates that indigenous or inoculated LAB are necessary for enhancing the antioxidant activity. The lactic acid fermentation process enhances the synthesis and enzymatic transformation of various bioactive compounds. The biotransformants and bacterial metabolites produced in this way increase antioxidant activity [38]. In addition to this mechanism, Zhang et al. [39] found that lactic acid stress increased the activity of antioxidant enzymes, including superoxide dismutase and glutathione peroxide, produced by *P. pentosaceus*. In this study, as TTA increased during kisra fermentation, such increased DPPH radical scavenging activity was probably attributed to lactic acid production by *P. pentosaceus* K-B21 and K-B01. Based on linear regression analysis, moderate positive correlations were indeed observed between TTA and the DPPH radical scavenging activity of the K-B21 (R^2^ = 0.65) and K-B01 (R^2^ = 0.81) groups (Figure S1). Furthermore, this increase in antioxidant activity could be attributed to the hydrolysis of more complex and massive phenolic compounds and the release of hydrolysates during fermentation, catalyzed by enzymes, such as glycosyl hydrolases, esterases, and tannases, derived from both flour and LAB [38]. In addition, LAB enzymes have a major effect in increasing the solubility of phenolic compounds through acidification [40]. Consistent with the results of the current study, Olojede et al. [41] reported that sorghum-based sourdough bread made using *P. pentosaceus* SA8 exhibited a similar level of DPPH scavenging activity as that in the control bread (without inoculation). Omedi et al. [42] also reported that the phenolic acid levels in samples fermented with LAB strains were similar to those in the control during fruit substrate fermentation, which is similar to the results obtained in this study. Therefore, the previous and current studies imply that not only the use of LAB starter cultures but also natural lactic acid fermentation may enhance the antioxidant activity of lactic acid fermented foods. It is also worth noting that the effect of lactic acid fermentation on the content of tannin, another antioxidant component, was evaluated, but no favorable changes caused by LAB were observed [38].

Beyond the effects of fermentation, the antioxidant indices significantly increase in all groups after baking. The increase in DPPH scavenging activity after baking in all groups is aligned with a study by Sidari et al. [43], who reported that both inoculated and non-inoculated breads showed higher DPPH scavenging activity after baking compared to their corresponding fermented doughs. Similarly, the increase in phenolic content after baking is consistent with the findings by Lu et al. [44], who reported that unbaked doughs generally had slightly lower phenolic content compared to baked breads. They suggested that this was likely due to increased bioaccessibility caused by intense heat. Moreover, the occurrence of the Maillard reaction during baking may contribute to the formation of new phenolic structures [45]. Taken together, lactic acid fermentation and baking may significantly contribute to the antioxidant activity of sourdough bread through the above mechanisms.

## 4. Conclusions

The presence of undesirable LAB during natural fermentation is responsible for the formation of BAs, such as histamine, tyramine, putrescine, and cadaverine. Conversely, fermentation with LAB enhances health functionality, such as antioxidant activity, through various mechanisms discussed in this study. Therefore, research on both the health functionality and safety of LAB and their use as industrial starter cultures are very important in the fermentation and food industries.

In the current study, it was demonstrated that applying *P. pentosaceus* K-B21 and *P. pentosaceus* K-B01, isolated from natural ly fermented kisra sourdough, with low BA-producing and high BA-degrading ability to fermentation is an effective method to reduce BA formation. That is, compared to naturally fermented kisra without inoculation (control group), *P. pentosaceus* K-B21 and *P. pentosaceus* K-B01 completely inhibited the formation of tyramine and cadaverine and significantly reduced the formation of histamine, putrescine, spermine, and spermidine by about 9 to 90%, depending on the type of BAs. In particular, *P. pentosaceus* K-B21, which had a higher BA-degrading activity, showed a slightly better inhibitory effect.

Meanwhile, the use of the two strains for kisra fermentation also enhanced the antioxidant indices of kisra, including DPPH radical scavenging activity and total phenolic content, with similar change patterns to natural fermentation (control). Particularly, *P. pentosaceus* K-B21-inoculated kisra bread had the highest DPPH scavenging activity of 84.17% among the three groups, including the control. Total phenolic content was also high at 1851.20 μg/g and 1880.60 μg/g in *P. pentosaceus* K-B21- and K-B01-inoculated kisra breads, respectively.

Taken together, using *P. pentosaceus* K-B21 (and K-B01 as well) in the fermentation of sourdough such as kisra inhibits BA formation and enhances the antioxidant indices, which suggests that industrial use of these LAB strains as starter cultures may greatly improve both the safety and health functionality of fermented foods, including sourdough breads such as kisra.

## Figures and Tables

**Table 1:** In vitro capabilities of producing and degrading BAs by LAB strains isolated from kisra sourdough.

BA-Related Metabolic Capabilities		LAB Strains Isolated from Kisra Sourdough		
		*P. pentosaceus* K-B21	*P. pentosaceus* K-B01	Other Strains (n = 44 ^1^)
**BA Production** **(μg/mL)**	HIS ^2^	ND ^3,b^	0.19 ± 0.01 ^4,a^	0.17 ± 0.17 ^5,a^
	TYR	0.69 ± 0.02 ^b^	0.63 ± 0.01 ^b^	142.55 ± 78.15 ^a^
	PUT	0.37 ± 0.01 ^a^	0.37 ± 0.01 ^a^	0.26 ± 0.08 ^a^
	CAD	0.08 ± 0.01 ^a^	0.08 ± 0.01 ^a^	0.14 ± 0.05 ^a^
**Degradation Rate** **(%)**	HIS	8.06 ± 5.86 ^a^ (3.91–12.20) ^6^	3.28 ± 4.64 ^b^ (0.00–6.56)	- ^7^
	TYR	8.38 ± 9.01 ^a^ (2.00–14.75)	6.20 ± 6.33 ^b^ (1.72–10.66)	-

^1^ The number of LAB strains screened based on BA production ability of each strain. ^2^ HIS: histamine, TYR: tyramine, PUT: putrescine, CAD: cadaverine. ^3^ ND: no production detected. ^4^ Values represent mean ± standard deviation measured in triplicate experiments. Mean values in the same column of the same BA followed by the same letter are not significantly different. ^5^ The mean ± standard deviation of BA production of multiple strains was calculated using the average BA production of each strain determined from biological triplicate experiments. ^6^ Values represent the mean ± standard deviation (minimum and maximum) as determined by triplicate experiments. ^7^ In vitro degradation tests for the other 44 strains were not determined.

## Data Availability

Data are contained within the article or Supplementary Material.

## Supplementary Materials

The following supporting information can be downloaded at: https://www.mdpi.com/article/10.3390/antiox13101204/s1, Table S1: Changes in physicochemical and microbial properties during kisra fermentation with or without *P. pentosaceus* strains; Table S2: Changes in biogenic amine content during kisra fermentation with or without *P. pentosaceus* strains; Table S3: Changes in antioxidant indices during kisra fermentation with or without *P. pentosaceus* strains; Figure S1: The linear regression between total titratable acidity and DPPH radical scavenging activity of kisra fermented with the selected *P. pentosaceus* strains. (a) Control group (kisra naturally fermented without an inoculum), (b) K-B21 group (kisra fermented with *P. pentosaceus* K-B21), (c) K-B01 group (kisra fermented with *P. pentosaceus* K-B01). Each dot indicates the mean value determined at each period of kisra processing.
