# Supplementary material for: Influence of Pediococcus pentosaceus Starter Cultures on Biogenic Amine Content and Antioxidant Activity in African Sourdough Flatbread Fermentation
Source: Antioxidants (Basel). 2024 Oct 7;13(10):1204. doi: 10.3390/antiox13101204 (PMC11504419; doi:10.3390/antiox13101204)
Supplement: Supplementary file 1 [file antioxidants-13-01204-s001.zip › 6.0-LAB for kisra bread-Antioxidants-proofreading-Sup-FIgure.pdf]

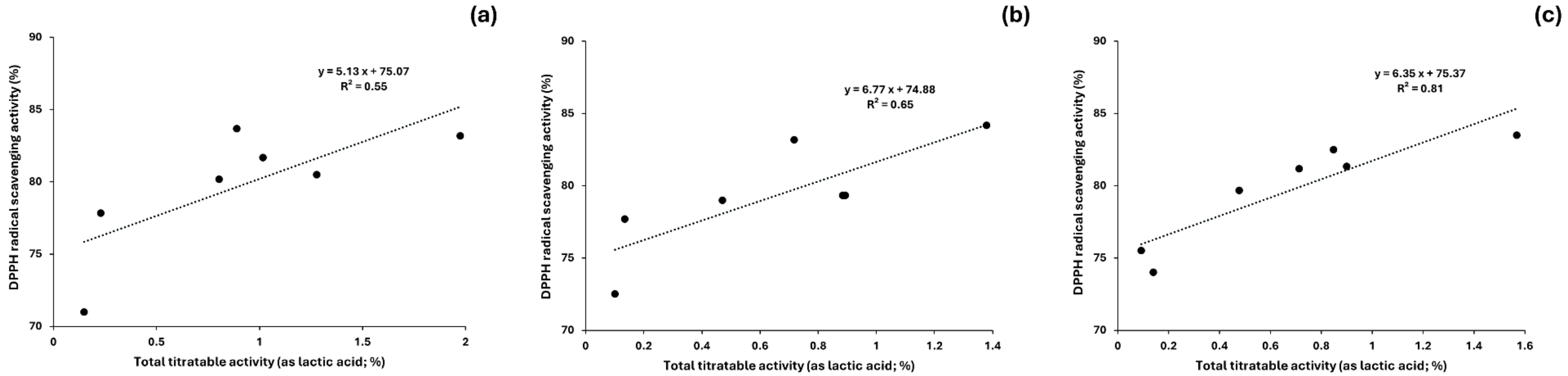

Figure S1. The linear regression between total titratable acidity and DPPH radical scavenging activity of kisra fermented with the selected *P. pentosaceus* strains. (a) Control group (kisra naturally fermented without an inoculum), (b) K-B21 group (kisra fermented with *P. pentosaceus* K-B21), (c) K-B01 group (kisra fermented with *P. pentosaceus* K-B01). Each dot indicates the mean value determined at each period of kisra processing.
