# Supplementary material for: Influence of Pediococcus pentosaceus Starter Cultures on Biogenic Amine Content and Antioxidant Activity in African Sourdough Flatbread Fermentation
Source: Antioxidants (Basel). 2024 Oct 7;13(10):1204. doi: 10.3390/antiox13101204 (PMC11504419; doi:10.3390/antiox13101204)
Supplement: Supplementary file 1 [file antioxidants-13-01204-s001.zip › 6.0-LAB for kisra bread-Antioxidants-proofreading-Sup-Table.pdf]

Table S1. Changes in physicochemical and microbial properties during kisra fermentation with or without *P. pentosaceus* strains.

Groups [1]	Physicochemical and microbial properties	Processing stages						
		First fermentation			Second fermentation			Baked
		0 h	24 h	48 h	0 h	6 h	12 h	kisra
**Blank group**	LAB count (log CFU/g)	ND [2]	ND	ND	ND	ND	ND	ND
	pH	6.89±0.01 [3] ^{a}	6.90±0.01 ^{a}	6.89±0.01 ^{a}	6.91±0.01 ^{a}	6.91±0.01 ^{a}	6.91±0.01 ^{a}	6.83±0.01 ^{b}
	a_w	0.988±0.001 ^{b}	0.988±0.001 ^{b}	0.988±0.001 ^{b}	0.991±0.001 ^{a}	0.991±0.001 ^{a}	0.991±0.001 ^{a}	0.978±0.001 ^{c}
	TTA (%) [4]	0.13±0.01 ^{b}	0.13±0.02 ^{b}	0.12±0.01 ^{b}	0.13±0.01 ^{b}	0.13±0.02 ^{b}	0.13±0.02 ^{b}	0.24±0.01 ^{a}
**Control group**	LAB count (log CFU/g)	6.00±0.08 ^{c}	9.12±0.21 ^{a}	9.06±0.09 ^{a}	7.93±0.05 ^{b}	9.13±0.00 ^{a}	9.19±0.01 ^{a}	ND ^{d}
	pH	7.03±0.01 ^{a}	4.28±0.36 ^{b}	3.76±0.05 ^{b}	6.15±0.04 ^{a}	3.98±0.02 ^{b}	3.78±0.00 ^{b}	3.64±0.00 ^{b}
	a_w	0.987±0.000 ^{a}	0.987±0.002 ^{a}	0.983±0.004 ^{a}	0.984±0.004 ^{a}	0.984±0.000 ^{a}	0.985±0.000 ^{a}	0.973±0.002 ^{b}
	TTA (%) [4]	0.15±0.01 ^{e}	0.89±0.17 ^{cd}	1.28±0.03 ^{b}	0.23±0.00 ^{e}	0.80±0.06 ^{d}	1.02±0.03 ^{c}	1.98±0.00 ^{a}
**K-B21 group**	LAB count (log CFU/g)	7.33±0.08 ^{e}	9.00±0.00 ^{ab}	8.37±0.13 ^{c}	7.59±0.10 ^{d}	8.91±0.03 ^{b}	9.12±0.08 ^{a}	ND ^{f}
	pH	6.85±0.05 ^{a}	3.87±0.01 ^{e}	3.79±0.00 ^{f}	6.08±0.03 ^{b}	4.40±0.02 ^{c}	3.99±0.00 ^{d}	3.82±0.01 ^{ef}
	a_w	0.988±0.007 ^{a}	0.991±0.002 ^{a}	0.985±0.002 ^{ab}	0.990±0.004 ^{a}	0.984±0.000 ^{ab}	0.981±0.001 ^{bc}	0.975±0.000 ^{c}
	TTA (%)	0.10±0.00 ^{e}	0.88±0.05 ^{b}	0.86±0.08 ^{b}	0.14±0.00 ^{e}	0.47±0.05 ^{d}	0.72±0.03 ^{c}	1.38±0.01 ^{a}
**K-B01 group**	LAB count (log CFU/g)	6.88±0.14 ^{c}	9.05±0.04 ^{c}	8.39±0.04 ^{ab}	8.08±0.88 ^{b}	9.00±0.033 ^{a}	9.08±0.03 ^{a}	ND ^{d}
	pH	6.88±0.00 ^{a}	3.85±0.00 ^{e}	3.80±0.00 ^{f}	6.08±0.01 ^{b}	4.40±0.04 ^{c}	4.00±0.01 ^{d}	3.81±0.01 ^{ef}
	a_w	0.988±0.006 ^{ab}	0.986±0.003 ^{ab}	0.987±0.001 ^{ab}	0.989±0.001 ^{a}	0.984±0.001 ^{ab}	0.983±0.003 ^{ab}	0.979±0.010 ^{b}
	TTA (%)	0.09±0.00 ^{e}	0.85±0.03 ^{bc}	0.90±0.03 ^{b}	0.14±0.00 ^{e}	0.48±0.03 ^{d}	0.71±0.05 ^{c}	1.57±0.14 ^{a}

[1] Blank group (kisra non-fermented), control group (kisra naturally fermented without an inoculum), K-B21 group (kisra fermented with *P. pentosaceus* K-B21), K-B01 group (kisra fermented with *P. pentosaceus* K-B01).

[2] ND: not detected.

[3] Data represented as mean±standard deviation from triplicate measurements. Values in the same row followed by different letters (a-f) are significantly different ($p < 0.05$).

[4] Total titratable acidity expressed as lactic acid percentage.

Table S2. Changes in biogenic amine content during kisra fermentation with or without *P. pentosaceus* strains.

Groups [1]	Processing stages		Biogenic amine content (mg/kg) [2]							
			TRP	PHE	PUT	CAD	HIS	TYR	SPD	SPM
Blank group	First Fermentation	0 h	ND [3]	ND	2.23±0.17 [4,ab]	ND	3.95±0.23 [a]	ND	5.16±0.05 [c]	5.95±0.10 [c]
		24 h	ND	ND	2.17±0.08 [b]	ND	3.61±0.08 [b]	ND	5.10±0.03 [c]	5.95±0.04 [c]
		48 h	ND	ND	2.18±0.04 [b]	ND	3.68±0.13 [ab]	ND	5.09±0.01 [c]	5.99±0.06 [c]
	Second Fermentation	0 h	ND	ND	1.82±0.09 [c]	ND	3.05±0.09 [c]	ND	5.79±0.02 [b]	6.47±0.12 [b]
		6 h	ND	ND	1.61±0.21 [c]	ND	3.11±0.13 [c]	ND	5.80±0.04 [b]	6.42±0.09 [b]
		12 h	ND	ND	1.67±0.16 [c]	ND	3.12±0.05 [c]	ND	5.79±0.01 [b]	6.33±0.16 [b]
	Baked	kisra	ND	ND	2.52±0.06 [a]	ND	3.35±0.18 [bc]	ND	6.80±0.04 [a]	8.16±0.21 [a]
Control group	First Fermentation	0 h	ND	ND	2.32±0.44 [d]	ND [e]	2.00±0.43 [d]	ND [g]	7.38±1.55 [b]	4.65±0.11 [cd]
		24 h	ND	ND	96.00±4.78 [b]	135.08±6.08 [b]	4.16±0.05 [c]	42.40±0.63 [b]	7.60±0.46 [b]	3.24±0.14 [e]
		48 h	ND	ND	127.81±24.38 [a]	158.45±9.73 [a]	39.68±0.47 [a]	68.21±1.28 [a]	8.02±0.22 [b]	3.89±0.59 [de]
	Second Fermentation	0 h	ND	ND	13.34±0.23 [cd]	12.58±0.61 [d]	2.24±1.21 [d]	6.64±0.69 [f]	6.82±0.23 [b]	4.77±0.15 [cd]
		6 h	ND	ND	17.02±1.08 [cd]	15.08±0.85 [cd]	4.18±0.17 [c]	17.65±0.44 [c]	7.01±1.12 [b]	5.16±0.03 [c]
		12 h	ND	ND	14.53±1.15 [cd]	15.31±2.91 [cd]	4.08±0.10 [c]	8.55±0.49 [e]	7.23±0.56 [b]	7.71±0.20 [b]
	Baked	kisra	ND	ND	25.40±5.18 [c]	25.68±0.78 [c]	7.78±0.81 [b]	12.39±0.03 [c]	10.46±0.04 [a]	9.42±1.18 [a]
K-B21 group	First Fermentation	0 h	ND	ND	2.01±0.17 [a]	ND	4.72±0.17 [ab]	ND	5.01±0.22 [de]	5.03±0.55 [a]
		24 h	ND	ND	2.57±0.50 [a]	ND	3.39±0.10 [bc]	ND	4.80±0.10 [e]	2.32±0.02 [b]
		48 h	ND	ND	2.95±0.28 [a]	ND	3.24±0.36 [c]	ND	5.51±0.51 [cde]	2.07±0.91 [b]
	Second Fermentation	0 h	ND	ND	1.46±0.03 [a]	ND	3.81±0.06 [abc]	ND	6.06±0.30 [cd]	5.38±0.33 [a]
		6 h	ND	ND	1.69±0.29 [a]	ND	3.88±0.16 [abc]	ND	6.56±0.73 [bc]	4.48±0.53 [a]
		12 h	ND	ND	1.46±2.06 [a]	ND	5.05±0.51 [a]	ND	7.49±0.32 [b]	2.60±0.08 [b]
	Baked	kisra	ND	ND	2.66±0.98 [a]	ND	5.11±1.36 [a]	ND	9.50±0.79 [a]	4.24±0.68 [a]
K-B01 group	First Fermentation	0 h	ND	ND	2.00±0.29 [bcd]	ND	5.10±0.23 [a]	ND	5.01±0.30 [de]	2.67±0.21 [bc]
		24 h	ND	ND	3.02±0.16 [a]	ND	3.89±0.16 [bc]	ND	4.89±0.53 [e]	2.07±0.01 [c]
		48 h	ND	ND	2.92±0.12 [a]	ND	3.16±0.19 [c]	ND	5.70±1.09 [cde]	1.70±0.27 [c]
	Second Fermentation	0 h	ND	ND	1.50±0.15 [d]	ND	3.96±0.19 [bc]	ND	6.14±0.03 [bcd]	4.83±0.50 [a]
		6 h	ND	ND	2.06±0.19 [bc]	ND	4.10±0.63 [bc]	ND	7.00±0.10 [ab]	3.48±1.05 [b]
		12 h	ND	ND	1.96±0.40 [cd]	ND	4.46±0.57 [ab]	ND	6.77±0.53 [abc]	2.21±0.15 [c]
	Baked	kisra	ND	ND	2.53±0.11 [ab]	ND	5.32±0.59 [a]	ND	7.90±0.10 [a]	3.67±0.14 [b]

[1] Blank group (kisra non-fermented), control group (kisra naturally fermented without an inoculum), K-B21 group (kisra fermented with *P. pentosaceus* K-B21), K-B01 group (kisra fermented with *P. pentosaceus* K-B01).

[2] TRP: tryptamine; PHE: β-phenylethylamine; PUT: putrescine; CAD: cadaverine; HIS: histamine; TYR: tyramine; SPD: spermidine; SPM: spermine.

[3] ND: not detected.

[4] Data represented as mean±standard deviation from triplicate measurements. Values in the same column of the same group followed by different letters (a-g) are significantly different ($p < 0.05$).

Table S3. Changes in antioxidant indices during kisra fermentation with or without *P. pentosaceus* strains.

Groups [1]	Processing stages		Antioxidant indices	
			**DPPH (%)**	**TPC (μg/g) [2]**
**Blank group**	**First Fermentation**	**0 h**	70.88±0.87 [3]^{b}	1325.10±19.72 ^{bc}
		**24 h**	70.93±0.83 ^{b}	1337.73±16.67 ^{bc}
		**48 h**	70.70±0.94 ^{b}	1341.37±11.30 ^{b}
	**Second Fermentation**	**0 h**	70.74±0.74 ^{b}	1321.17±25.42 ^{bc}
		**6 h**	70.52±0.47 ^{b}	1309.62±19.82 ^{c}
		**12 h**	70.22±0.59 ^{b}	1319.64±18.61 ^{bc}
	**Baked**	**kisra**	74.47±0.65 ^{a}	1522.24±19.81 ^{a}
**Control group**	**First Fermentation**	**0 h**	71.00±7.10 ^{b}	1230.60±58.20 ^{c}
		**24 h**	83.67±0.50 ^{a}	1301.20±49.90 ^{bc}
		**48 h**	80.50±2.60 ^{a}	1395.30±8.30 ^{b}
	**Second Fermentation**	**0 h**	77.83±0.20 ^{ab}	1274.70±37.40 ^{bc}
		**6 h**	80.17±2.60 ^{a}	1312.90±74.90 ^{bc}
		**12 h**	81.67±0.50 ^{a}	1257.10±112.30 ^{bc}
	**Baked**	**kisra**	83.17±1.20 ^{a}	1977.60±25.00 ^{a}
**K-B21 group**	**First Fermentation**	**0 h**	72.50±0.20 ^{c}	1321.80±4.02 ^{b}
		**24 h**	79.33±0.00 ^{ab}	1233.50±45.80 ^{b}
		**48 h**	79.33±2.80 ^{ab}	1248.20±83.20 ^{b}
	**Second Fermentation**	**0 h**	77.67±4.20 ^{bc}	1230.60±66.60 ^{b}
		**6 h**	79.00±3.30 ^{ab}	1233.50±54.10 ^{b}
		**12 h**	83.17±0.70 ^{a}	1342.40±25.00 ^{b}
	**Baked**	**kisra**	84.17±0.20 ^{a}	1851.20±70.70 ^{a}
**K-B01 group**	**First Fermentation**	**0 h**	75.50±0.70 ^{d}	1374.70±4.20 ^{b}
		**24 h**	82.50±0.20 ^{ab}	1351.20±4.20 ^{b}
		**48 h**	81.33±0.00 ^{bc}	1162.90±137.30 ^{c}
	**Second Fermentation**	**0 h**	74.00±0.94 ^{d}	1145.30±29.10 ^{c}
		**6 h**	79.67±1.41^{c}	1289.40±33.30 ^{bc}
		**12 h**	81.17±0.70 ^{bc}	1236.50±74.90 ^{bc}
	**Baked**	**kisra**	83.50±0.20 ^{a}	1880.50±128.90 ^{a}

[1] Blank group (kisra non-fermented), control group (kisra naturally fermented without an inoculum), K-B21 group (kisra fermented with *P. pentosaceus* K-B21), K-B01 group (kisra fermented with *P. pentosaceus* K-B01).
[2] Total phenolic content is expressed as μg of gallic acid equivalent/g of wet samples.
[3] Data represented as mean±standard deviation from triplicate measurements. Values in the same column of the same group followed by different letters (a-d) are significantly different ($p < 0.05$).
